# Periodic assessment of plasma sFlt-1 and PlGF concentrations and its association with placental morphometry in gestational hypertension (GH) - a prospective follow-up study

**DOI:** 10.1186/1471-2393-10-58

**Published:** 2010-09-28

**Authors:** Kamalan Jeevaratnam, Vishna Devi Nadarajah, John Paul Judson, Sivalingam Nalliah, Mohd Farouk Abdullah

**Affiliations:** 1 Department of Physiology, Development and Neuroscience, University of Cambridge, CB2 3EG Cambridge, UK; 2 Department of Human Biology, Faculty of Medicine, International Medical University, No.126, Jalan 19/155B Bukit Jalil, 57000 Kuala Lumpur, Malaysia; 3 Division of Human Development and Population Health, Faculty of Medicine, International Medical University, Jalan Rasah, 70300 Seremban, Negeri Sembilan, Malaysia; 4 Department of Obstetrics and Gynaecology, Tengku Ampuan Rahimah General Hospital, 41200, Klang, Selangor, Malaysia

## Abstract

**Background:**

Hypertensive disorders in pregnancy contributes to about 12% of maternal deaths in Malaysia and similarly worldwide. Early detection and adequate management are preventable strategies. Biochemical markers of abnormal angiogenesis would be more specific in early detection than routine blood pressure and proteinuria measurements. The aim of this study was to estimate maternal plasma PlGF and sFlt-1 levels in pregnant women with gestational hypertension at three intervals of pregnancy and correlate these biomarker levels with placental morphometry.

**Methods:**

Venous blood samples (antepartum, intrapartum and post partum periods) were drawn to estimate for sFlt-1 and PlGF levels while placental tissue samples were examined for placental morphometry.

**Results:**

PlGF levels were lower in gestational hypertension (GH) compared to normotensive during antepartum and intrapartum period, whereas sFlt-1 levels were elevated in GH at antepartum, intrapartum and postpartum intervals during pregnancy. An inverse relationship between these two biomarkers was observed through correlation analysis. PlGF levels were inversely correlated with total villous surface area of the placental periphery (TCsa-C) and villous capillarization (VC-C) of the placental periphery.

**Conclusion:**

We established periodic values of for sFlt-1 and PlGF levels for the first time in an ethnically diverse Malaysian setting. We suggest the development of GH in women is related to defective capillarization. In demonstrating periodic changes, this study suggest the possibility of developing GH and other long term health complications as a result of prolonged exposure to sFlt-1. The correlation between PlGF levels and morphometric findings also support possible capillarization defect.

## Background

Pregnancy Induced Hypertension which encompasses gestational hypertension (GH), preeclampsia and eclampsia contributes 12% of this total maternal mortality, thus highlighting the impact this condition has on a nation's healthcare system [1]. Of the 966 maternal deaths reported in Malaysia between 1997-2000 (Maternal Mortality Rate was 28.1 per 100 000 live births in 2000) hypertensive disorders of pregnancy accounted for 14.2% [2].

It has been proposed that placental angiogenesis is defective in preeclampsia, as evidenced by failure of the cytotrophoblast to convert from an epithelial to an endothelial phenotype (referred to as pseudovasculogenesis) thus causing variable degree of invasion of the maternal spiral arteries [3]. Furthermore, placenta isolated from women with early onset preeclampsia was associated with abnormal morphology compared to those with late onset preeclampsia [4] which suggests that placental ischemia is an early event relating to maternal endothelial dysfunction [5]. Recently serum soluble Fms-like tyrosine kinase 1 (sFlt1) expression has been noted to be elevated in preeclampsia [4]. sFlt-1 acts as an antagonist to pro-angiogenic factors Vascular Endothelial Growth Factor (VEGF) and Placental Growth Factor (PlGF) as it binds both VEGF and PlGF, thus reducing free circulating levels of these factors [5]. Maynard *et al*. (2003) demonstrated that excess sFlt1 in patients with preeclampsia causes endothelial dysfunction and produces a syndrome of nephrotic range characterized by proteinuria, hypertension and glomerular endotheliosis when administered in rats [5]. The effects circulating factors like sFlt1 and PlGF exert on placental structural changes and vasculopathies remains unclear although raised levels of these biomarkers have frequently been associated with the presence of the placenta.

While many studies have undertaken to measure these biomarkers in cases of preeclampsia, its clinical utility in GH has never been clearly defined and warrants further study. Women with new-onset, nonproteinuric hypertension after 20 weeks of gestation are provisionally diagnosed as GH, however there is significant risk for the development of preeclampsia or chronic hypertension [6]. A recent study by Khalil et al., 2008, suggests that pathophysiology of GH and preeclampsia are different as treatment with antihypertensive drugs had an effect on levels of sFlt-1 and soluble endoglin in preeclamptic groups compared to GH group, although these biomarkers were significantly raised in untreated GH [7]. Furthermore, most published work on these biomarkers have not attempted to correlate this values to that of placental structural changes. This study aims to compare maternal PlGF and sFlt-1 levels in GH mothers periodically (antepartum, intrapartum and postpartum) and later correlate these biomarker levels with placental morphometry. We hypothesize that there is periodic differences in sFlt1 and PlGF levels in GH and normotensive women and that there is a correlation between morphometry and biomarkers in these women.

## Methods

### Recruitment

Pregnant women with gestational age between 24 to 32 weeks were initially recruited to participate in this study from July 2006 till Feb 2007. During this period, participants were recruited for the study from Government Maternal and Child Health Clinic, Klang. Prior to recruitment, the prospective follow-up nature of the study and samples required were explained to all subjects and informed consent obtained. Sample collection for blood was done at antepartum (24-32 weeks), intrapartum (point of delivery) and at postpartum (6 week postpartum check-up). Participants were informed that they could choose to voluntarily withdraw from the study at any point. This research project was approved by the Research and Ethics Committee of the International Medical University (Res No: 113/2006). For this study, Gestational Hypertension (GH), was clinically defined as having blood pressure of ≥ 140/90 mm Hg on more than two occasions greater than 6 hours apart; without proteinuria after 20 weeks of pregnancy [8]. Patients suffering from essential hypertension, any type of renal disease, diabetes mellitus, heart diseases or infectious disease were excluded from this study. Women with incidence of IUGR and a history of smoking were also excluded.

### Biomarkers Studies

For plasma Placental Growth Factor (PlGF) levels quantification, a Human PlGF DuoSet ELISA Development Kit, DY 264 from R&D Systems Inc., Minneapolis, USA was used. It used the quantitative sandwich enzyme immunoassay technique where only free, unbound forms of the growth factor are detected. The intra-assay precision based on co-efficient of variations was 5.1% while inter-assay precision was 7% for the PlGF kit. For sFlt-1 analysis, all required chemicals and reagents were provided in the sFlt-1 ELISA kit by the same manufacturer (Human Soluble VEGF R1/Flt-1 Immunoassay, DVR100B, R&D Systems, Minneapolis, USA). The materials were sufficient for six 96-well microplates. Manufacturers' protocol was adhered to strictly and both kits were compatible for plasma analysis. This kit had an average intra and inters-assay precision of 3.2% and 7.4% respectively.

### Placental Studies

Three whole thickness placental sections, measuring about 1 cm^3 ^each were randomly taken from the central (A), body/middle (B) and periphery (C) of the placenta and fixed in formalin. Tissues were subsequently transported to the laboratory for routine tissue processing and paraffin blocking. Tissue blocks were sectioned at 5 μm thickness and stained with Hematoxylin and Eosin. Two dimensional morphometric analysis was done using computer aided software (Image Pro Express v 4.5.1.3) on pictures taken using a five megapixel camera (Evolution MP Cooled, Media Cybernetics, Canada) attached to a microscope (Leica DMLS HC, Leica Microsystem, Germany). For morphometric calculation, two dimensional measurement techniques were developed after reviewing techniques used in a previously published report by Egbor *et al*., (2006) [9].

**Figure 1:**
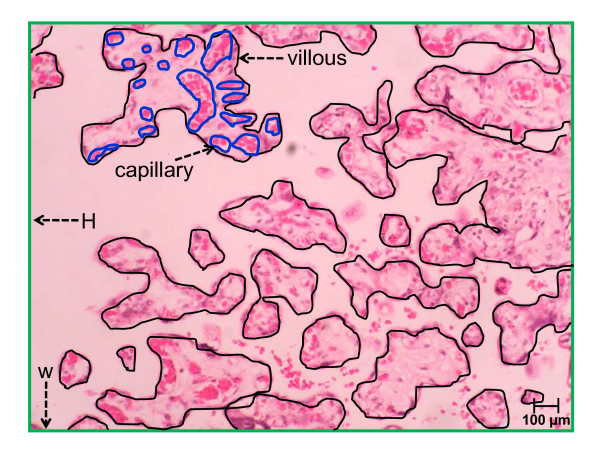
**Morphometric calculation for villous capillarization (VC) and intervillous space (IvS) of placental villi**. Calculation of VC was done by dividing the surface area of the capillary (blue outline) over the surface area of the villous (black outline). Calculation of IvS was done by subtracting the surface area of the photomicrograph (calculated by height {H} X width {W}; green outline indicated by black arrows) with the summed up area of all villous (black outline) within a particular photomicrograph. The surface area (mm^2^) of the respective capillaries and villous measured was generated by the software program. This slide was viewed at 20× magnification.

For the calculation of villous capillarization (VC), the total capillary surface area (TCsa) of a given primary villous is divided with its villous surface area to obtain the capillarization for that villous (Figure 1). In order to calculate the mean VC for one patient, three randomly selected primary villi from each of the three slides were used to obtain mean VC, i.e. a total of 9 primary villous from the three different location of the placenta were measured to obtain the mean VC. In this study, VC is representative of placental hypervascularity or hypovascularity.

For the calculation of the intervillous space (IvS), the total surface area of all the villous (TVsa) and its associated structures occupying a photomicrograph are summed up and then subtracted from the total surface area of the photomicrograph (Figure 1). In order to calculate the mean IvS for one patient, three randomly captured photomicrographs from each of the three slides were calculated to obtain mean IvS. For each patient, a total of 9 photomicrographs from three different locations of the placentas were captured to obtain the mean IvS. IvS calculation in this study was a representative of villous crowding. For the purpose of this study, villous crowding was defined as a pathological change in which there is a reduction in intervillous space associated with defective capillarization.

### Statistical Analysis

Statistical analyses were performed using the Statistical Package for the Social Sciences (SPSS), version 16. All data sets were subjected to normality testing using the Kolmogorov-Smirnov method. For comparisons between means the analysis of variance (ANOVA) was applied while the Spearman's Rank Correlation was used for correlation studies. The data was analyzed at 95% confidence interval and a P value < 0.05 was considered significant.

## Results

During the period of 1^st ^August 2006 till 1^st ^May 2007, a total of 162 pregnant women (117 normotensives and 45 Gestational Hypertension (GH)) were recruited for this study from the Government Maternal and Child Health Clinic, Klang and Tengku Ampuan Rahimah General Hospital Klang, Selangor Darul Ehsan. Table 1 describes the clinical and demographic information of the study population. Blood pressure during the three study intervals for normotensive women ranged from 107/69 mmHg to 112/72 mmHg whereas for GH women it ranged from 123/81 mmHg to 147/91 mmHg. Women from the Malay ethnicity represented the most number recruited by ethnicity and women in the 1-4 parity was similarly the highest represented group. Women recruited in the study were mostly between the age groups 25-28 and 29-32. Prior to normotensive and GH comparison, the data was analyzed for confounding effects of ethnicity, parity and maternal age. No significant differences were observed for all races, parity and maternal age in the normotensive and GH women for both biomarkers and placental morphometry thus allowing the grouping of the study population by normotensive and GH only.

**Table 1:** Clinical and demographic description of study population

	Average Blood Pressure ± SE (mmHg)						PROTEINURIA	RACE			PARITY		MATERNAL AGE (years)					
			
	Antepartum		Intrapartum		Postpartum			Malay	Chinese	Indian	0	1 - 4	> 5	17 - 24	25 - 28	29 - 32	33 - 36	37 - 44
	Systolic	Dystolic	Systolic	Dystolic	Systolic	Dystolic												
**Normotensive**	112 ± 1	72 ± 1	120 ± 2	74 ± 1	107 ± 3	69 ± 2	Nil	59	21	37	51	65	1	23	41	36	13	4

**GH**	143 ± 2	90 ± 2	147 ± 3	91 ± 2	123 ± 3	81 ± 2	Nil	30	5	10	18	22	5	5	13	11	9	7

SE = Standard Error

mmHg = milimetres of mercury

### Placental Growth Factor (PlGF)

**Figure 2:**
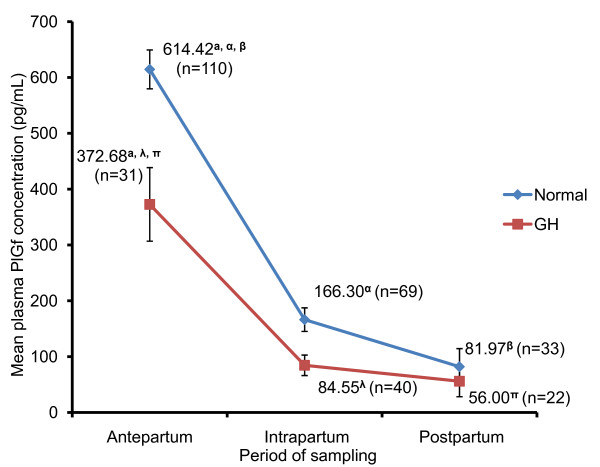
**Baseline levels of plasma PlGF in women based on period of sampling**. Baseline levels of plasma PlGF were determined using ELISA. Values displayed are mean ± standard error (SE) of the three intervals (antepartum, intrapartum and postpartum), obtained from normotensive and GH women. Means with similar superscript differ significantly at P < 0.05.

As depicted in Figure 2, biomarker analysis for PlGF revealed that the mean plasma concentration for normotensive women at 614.42 ± 34.79 pg/mL (n = 110) were significantly higher (P = 0.001), compared to GH women at 372.68 ± 65.86 pg/mL (n = 31) during antepartum. PlGF levels at intrapartum for normotensive (n = 69) and GH (n = 40) were found to be 166.30 ± 21.10 pg/mL and 84.55 ± 18.38 pg/mL respectively and were significantly different (P = 0.008). Postpartum PlGF levels in normotensive women (n = 33) were 81.97 ± 32.43 pg/mL and for GH (n = 22) women were 56.00 ± 27.78 pg/mL respectively and were statistically indifferent.

When analyzed by period of sampling, antepartum plasma PlGF concentration in normotensive women were significantly higher than plasma PlGF concentration during the intrapartum (P = 0.000) and postpartum (P = 0.000). Plasma PlGF concentration during intrapartum was however not significantly different from the postpartum period (P = 0.173). In GH patients, both intrapartum (P = 0.000) and postpartum (P = 0.000) plasma PlGF concentration were significantly lower than antepartum concentration respectively. No significant plasma PlGF concentration difference was observed between intrapartum and postpartum for GH women.

### Soluble Fms-like Tyrosine Kinase-1 (sFlt-1)

**Figure 3:**
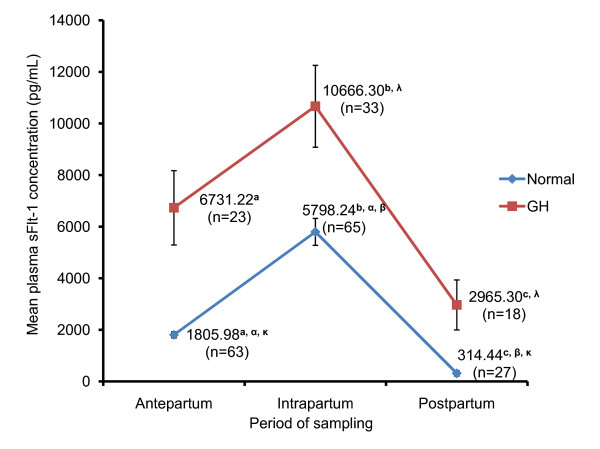
**Baseline levels of plasma sFlt-1 in women based on period of sampling**. Baseline levels of plasma sFlt-1 were determined using ELISA. Values displayed are mean ± standard error (SE) of the three intervals (antepartum, intrapartum and postpartum), obtained from normotensive and GH women. Means with similar superscript differ significantly at P < 0.05.

The mean plasma concentration as outlined in Figure 3 showed that sFlt-1 during the antepartum interval, for GH women (n = 23) at 6731.22 ± 1440.14 pg/mL was significantly higher than normotensive women (n = 63) at 1805.98 ± 120.59 pg/mL; P = 0.000. During the intrapartum period, GH women (n = 33) also showed significantly higher, plasma sFlt-1 concentration of 10666.30 ± 1587.90 pg/mL compared to normotensive women (n = 65) at 5798.24 ± 522.99 pg/mL, (P = 0.000). Postpartum plasma sFlt-1 concentration were significantly higher in GH women (n = 18) compared to normotensive women (n = 27) (GH - 2965.39 ± 969.68 pg/mL; Normotensive - 341.44 ± 107.83 pg/mL, P = 0.002).

It was also observed that in normotensive women sFlt-1 concentration during intrapartum was significantly higher than sFlt-1 concentration during antepartum (P = 0.000) and postpartum (P = 0.000) sampling period. Antepartum sFlt-1 concentration was also found to be significantly higher when compared to postpartum sFlt-1 (P = 0.025) concentration. In GH women however, antepartum sFlt-1 concentration was not significantly different in comparison to intrapartum (P = 0.058) and postpartum (P = 0.115) sFlt-1 concentrations. The intrapartum sFlt-1 concentration was noted to be significantly higher than the postpartum sFlt-1 (P = 0.001) concentration.

### Biomarker Correlation Analysis

For both normotensive and GH, biomarker correlation showed that sFlt-1 levels during antepartum was found to be positively correlated to sFlt1 levels during intrapartum (rho = 0.321; P = 0.002) but inversely related to sFlt-1 levels during postpartum (rho = -0.552; P = 0.000) and PlGF levels during antepartum (rho = - 0.216; P = 0.040) in the women studied for this research. It was also observed that sFlt-1 levels during intrapartum was inversely related to PlGF antepartum (rho = -0.266; P = 0.011). PlGF levels during antepartum was found to be significantly correlated to PlGF levels during intrapartum (rho = 0.449; P = 0.000). All other correlations were found to be not significant.

### Morphometric results for villous capillarization (VC) and intervillous space (IvS)

When placental weight was measured, there were no significant difference observed between both normotensive and GH women and the study thus proceeded to microscopic determination. As shown in Table 2, it was observed that the ratio for central villous capillarization (VC-A) for normotensive women was lower than GH women but were statistically insignificant. Similarly, villous capillarization for the placental body (VC-B) and placenta periphery (VC-C) were not significantly different between normotensive and GH women. IvS calculations observed that the central intervillous space (IvS-A), intervillous space for placental body (IvS-B) and intervillous space for placental periphery (IvS-C) was marginally higher in normotensive compared to GH but were not significantly different.

**Table 2:** Morphometric results for placental villous capillarisation and intervillous space

	Villous capillarisation (VC ± SE)			Intervillous Space (IvS ± SE, μm)		
	
	Central (VC-A)	Body (VC-B)	Periphery (VC-C)	Central (IvS-A)	Body (IvS-B)	Periphery (IvS-C)
**Normotensive** **(n = 51)**	0.12 ± 0.014	0.11 ± 0.007	0.12 ± 0.013	58778 ± 1723.64	59015.72 ± 1496.93	61518.28 ± 2479.36

**GH** **(n = 32)**	0.15 ± 0.014	0.13 ± 0.015	0.11 ± 0.015	57241.99 ± 1650.84	58380.34 ± 2753.76	55701.89 ± 1303.06

No significant differences were observed between means at P < 0.05

### Correlation of morphometry and biomarker

For both normotensive and GH, total villous surface area for central placenta (TVsa-A) was observed to be inversely correlated to sFlt-1 at antepartum (rho = -0.308; P = 0.014), but was however positively correlated with PlGF levels during antepartum (rho = 0.340; P = 0.005). VC-A was positively correlated to sFlt-1 levels during antepartum (rho = 0.264; P = 0.037). Total capillary surface area of the placental periphery (TCsa-C) was found to be inversely related to PlGF levels during postpartum (rho = -0.370; P = 0.019) and VC - C was also inversely related to PlGF levels during postpartum (rho = -0.364; P = 0.021). IvS-A was positively correlated to sFlt-1 levels during postpartum (rho = 0.439; P = 0.007) while IvS-B was inversely correlated to PlGF levels during antepartum (rho = -0.270; P = 0.026)

## Discussion

While there has been an increase in interest on the role circulating pro and anti-angiogenic factors play in the pathophysiology of GH, less is known on how these biomarkers vary over the duration of pregnancy and after delivery. This is particularly true in Malaysia where the incidence of GH is relatively high [10] but biomarker screening and follow up studies are limited. The present study accordingly performs three periodic assessments of both sFtl-1 and PlGF in GH women. It is further complemented with morphometric studies that allowed correlation of biomarker values and its role in placental structural development. Furthermore, this study emphasizes GH specifically; most published articles investigated the use of the biomarkers (PlGF and sFlt-1) for preeclampsia, a more serious condition superseding GH. Findings from this study also serve as a valuable reference point for an ethnically diverse population like Malaysia.

We observed lower PlGF but higher sFlt-1 levels in GH women in comparison to normotensive during antepartum and intrapartum, with correlation analysis confirming an inverse relationship. Salahudin *et al*., (2007) reported similar findings in that sFlt-1 levels in GH women were significantly higher than normotensive women in a prevalence case control study done in the United States [11]. Findings by Maynard *et al*., (2003) and Levine *et al*., (2005) both observed reduction in PlGF and an increase in sFlt-1 in preeclamptic women [5,12]. While the latter studies proposed this for preeclampsia, this study proves these biomarkers are also significantly altered in GH. Furthermore, the inverse relationship between sFlt-1 and PlGF observed in our study is representative of findings from an ethnically diverse population. We acknowledge that results from this study present a contrasting finding compared to a previous cross sectional study. In the latter study, while no absolute values of the biomarkers were discussed, analysis for sFlt1:PlGF ratios demonstrated no significant differences between GH and normotensive mothers except at 33 through 36 weeks of pregnancy [13]. This contrast could be due to variations in the study population (for example: age, parity, ethnicity, smoking status) or on the actual onset of GH versus the diagnosis of GH.

PlGF levels in GH and normotensive women during the postpartum period were similar and this can be attributed to the fact that PlGF is primarily expressed by the placenta which is no longer present at postpartum. Our study showed that sFlt-1 levels remain significantly higher in GH women compared to normotensive women during postpartum period. This finding raises important questions on the effect of persistently raised postpartum levels of sFlt-1, may have on the period of surveillance and continuation of anti-hypertensive drugs. Cohort studies that have used population-based pregnancy databases consistently identify a clinically significant association of GH and preeclampsia with later hypertensive disorders [14]. This clearly defines a need for long term follow-up for the development of essential hypertension in GH women. A recent study by Berks et al., 2009, on preeclamptic women in the Netherlands indicates that it can take up to 2 years for hypertension and proteinuria to resolve [15]. Findings from these studies suggest that it may be necessary to further evaluate the effect prolonged exposure to high levels of sFlt-1 may have and it's subsequent clinical utility in the management of GH cases.

The findings for villous capillarization and intervillous space were not significantly different among the two groups investigated (normotensive and GH). Our morphological findings were consistent with that of Mayhew *et al*., (2004) who reported that in cases of preeclampsia not accompanied with intrauterine growth restriction, placental morphometry is similar to that of control (normotensive) group [16]. It is hypothesized that, in GH cases there is increased anti-angiogenic property thus causing defective angiogenesis, in turn leading to decreased villous capillarization [5,12]. However, increased villous capillarization can also occur in GH women as a form of a compensatory mechanism. Hypoxic experiments with placental tissue have previously demonstrated an increase in vascular endothelial growth factor expressions [17]. These angiogenic factors may thus bind to receptors to exert their effect and promote angiogenesis by increasing capillarization explaining the lack of morphometric differences between normotensive and GH as seen in our study.

The inverse correlation PlGF levels show with TCsa - C and VC - C warrants further investigation. While is it expected that PlGF should increase capillarization, it needs to be ascertained whether this inverse relationship is a compensatory response, in that, when there is low capillarization, PlGF is released to promote capillarization thus explaining the scenario whereby the lower TCSA - C and VC - C is, the more PlGF is released. Future studies using animal models would be valuable in elucidating the existence of such a compensatory mechanism. There were several limitations that were present in this study. Shortfalls with regards to follow-up of patients from time of first contact was attributed to the fact that many women use the free government antenatal care services but prefer to deliver at alternative delivery centers which included returning to their parent's homes or to private medical centers when they were at term gestation. In the initial recruitment Body Mass Index (BMI) of patients were not obtained and thus we were unable to adjust for its confounding effects. Furthermore as this study examined two-dimensional evaluations of the placenta, future studies should include three-dimensional morphometric assessments with precise and in-depth structural and stereological features.

## Conclusion

Our work primarily studied biomarker values, placental morphometric parameters and their correlation in a Malaysian setting. In doing so, our findings represent an important source of information for a country with an ethnically diverse population and a maternal healthcare system that is undergoing rapid development. We therefore conclude that our study, establishes periodic values for both sFlt-1 and PlGF levels with placental morphometric correlations in a Malaysian setting and extends the idea that the development of GH in women assessed in this study is related to defective capillarization caused by an inverse relationship between the pro-angiogenic factor, PlGF and the anti-angiogenic factor, sFlt-1. These periodic biomarker levels suggest a clear need to evaluate the clinical utility of both sFlt-1 and PlGF, in managing GH from a surveillance point of view. The correlation between PlGF levels and morphometric findings indicate possible capillarization defect but larger cohort studies may be warranted.

## Abbreviations

GH: Gestational Hypertension; PlGF: Placental Growth Factor; sFlt-1: Soluble Fms-like Tyrosine Kinase-1; VEGF: Vascular Endothelial Growth Factor; (A): Placental central; (B): Placental body; (C): Placental periphery; VC: Villous capillarization; TCsa: Total capillary surface area; IvS: Intervillous space; TVsa: Total surface area of all the villous; VC-A: Central villous capillarization; VC-B: Villous capillarization for the placental body; VC-C: Villous capillarization for placenta periphery; IvS-A: Central intervillous space; IvS-B: Intervillous space for placental body; IvS-C: Intervillous space for placental periphery; TVSA-A: Total Villous Surface Area for Central Placenta; TCSA-C: Total capillary surface area of the placental periphery; mmHg: Millimetres of mercury; pg/mL: Picograms per millilitre; SE: Standard Error

## Competing interests

The authors declare that they have no competing interests

## Authors' contributions

KJ, VDN, JPJ, and SN contributed to the main idea of the project and were instrumental in the drafting of the research article. KJ and MFA were involved with the sample acquisition at the government hospital while KJ was involved in the completion of laboratory work. KJ, VDN and JPJ were involved with the assessment, analysis of data and interpretation of results. All authors contributed to the revision of the article and gave their approval of the final version.

## Author information

Dr Kamalan Jeevaratnam DAHP, DVM, MMedSc

Dr Vishna Devi Nadarajah BSc (Hons) (Biochem), PhD

Prof Dr John Paul Judson MBBS, DHA

Prof Dr Sivalingam Nalliah MBBS, MRCOG, FRCOG

Dr Farouk Abdullah MBBS, MRCOG, FRCOG

All authors in this article are Good Clinical Practice (GCP) certified researchers by the Ministry of Health, Government of Malaysia.

## Pre-publication history

The pre-publication history for this paper can be accessed here:

http://www.biomedcentral.com/1471-2393/10/58/prepub
